# The provision of additional services in primary care: a cross-sectional study of incentivised additional services, social deprivation, and ethnic group

**DOI:** 10.3399/bjgpopen20X101141

**Published:** 2021-01-06

**Authors:** Veline L'Esperance, Peter Schofield, Mark Ashworth

**Affiliations:** 1 School of Population Health & Environmental Sciences, King's College London, London, UK

**Keywords:** primary health care, general practice, health inequality, health status disparities

## Abstract

**Background:**

Primary care in England is contracted to provide essential services. Many practices also provide additional services, termed ‘directed enhanced services’ (DES), for extra income. The optional nature of DES may result in inequitable service delivery.

**Aim:**

To determine the range of DES activity and equity of service provision.

**Design & setting:**

A cross-sectional analysis of data from general practices in England took place from 2018–2019.

**Method:**

DES were defined in terms of activity level and measured as total DES funding per registered patient. Linear regression modelling was used to explore the relationship between DES activity, practice, and population characteristics.

**Results:**

Data were available for 6873 practices providing up to 10 DES in the initial sample. Due to negative funding amounts and a list size of ≤750 registered patients, 24 practices were excluded. Of the final sample (*n* = 6849), highest DES provision was for influenza and pneumococcal immunisation (99.9%), pertussis immunisation (97.9%), rotavirus and shingles immunisation (99.9%), meningitis immunisation (99.7%), and childhood immunisation (99.6%); lowest provision was for extended hours access (72.4%), violent patient services (2.0%), and out-of-area urgent care (1.3%). Mean DES funding was £6.25 per patient. In deprived areas, DES funding was £0.35 lower (95% confidence interval [CI] = £0.60 to £0.10) per patient (most versus least deprived quintiles); ethnic group-related differences were not significant. DES funding was higher in practices with more GPs or practice nurses per patient. In deprived communities, there was less immunisation activity (including influenza, pneumococcal, meningitis, childhood, and rotavirus and shingles immunisation) and provision of extended hours access; however, learning disability checks provision was greater in these communities.

**Conclusion:**

DES provision is lower in deprived areas (notably for immunisations and some aspects of access) but higher in better staffed practices. Voluntary quality schemes may contribute to widening health inequalities.

## How this fits in

There is a large amount of literature about the Quality and Outcomes Framework (QOF), but very little is published about another primary care performance-related payment, the provision of DES. Since the gradual reduction of QOF funding over the past decade, DES now account for a larger proportion of funding than QOF funding. The study demonstrates a mismatch between primary healthcare provision of DES and societal patterns of social deprivation and ethnic group. The mismatch raises questions about the funding of primary care and health equity. One solution would be to link performance-related pay to social deprivation.

## Introduction

In 2004, the UK government agreed a new contract with GPs that saw the introduction of the Quality and Outcomes Framework (QOF), the world’s largest pay-for-performance system in primary care, viewed as both a vehicle for improving the quality of primary care and for allocating additional incentive-based income.^1^ Less widely known is that at the same time, the contract allocated additional income for the provision of specified additional services in primary care.^2^

When introduced in 2004, nationally agreed schemes for providing additional primary care services covered services such as immunisations, extended opening hours, minor injury services, and many others.^3^ The scheme has now evolved and is termed 'directed enhanced services’ (DES), and in 2018–2019 covered 10 services (Table 1).^4^ Further development of the DES scheme in 2020–2021 aims to allocate funding to general practices for working in networks to collaborate in the achievement of specified population health initiatives, such as cardiovascular disease prevention, enhanced health care in care homes, or medication reviews to reduce polypharmacy in older people.^5^

**Table 1.** Summary of available DES and proportions of practices offering each service, *N* = 6849

**DES**	**Practices offering service, *n* (%)**
Influenza and pneumococcal immunisations	6844 (99.9)
Rotavirus and shingles immunisation	6839 (99.9)
Meningitis immunisation	6829 (99.7)
Childhood vaccination and immunisations	6820 (99.6)
Pertussis immunisation	6708 (97.9)
Learning disabilities health checks	5776 (84.3)
Minor surgery	5240 (76.5)
Extended hours access	4961 (72.4)
Violent patient services	134 (2.0)
Out-of-area, in-hours urgent care	89 (1.3)

DES = directed enhanced service.

Additional services were intended to address specific healthcare needs, both at a national or local level. Local schemes, initially termed ‘local enhanced services’ (LES), are now referred to as ‘local incentive services’ (LIS). Evaluating these schemes has proved challenging because there is no central register of services provided nor breakdown of funding.^6^

Concerns have been raised that both the capitation funding formula and QOF funding may result in widening health inequalities.^7–10^ Funding does not at present sufficiently reflect increased healthcare need experienced by older, more multimorbid, or more deprived populations.^7^ In 2018–2019, capitation and QOF funding sources accounted for 57.1% and 7.6%, respectively, of total primary care funding.^11^ In the same year, DES and LIS payments accounted for a larger proportion of total funding than the QOF at 4.2% and 5.2%, respectively.^11^ Both schemes are voluntary, with practices themselves making the decision whether to participate and accept the funding, or not to participate and relinquish the opportunity for funding. Factors that influence uptake of voluntary funding schemes are likely to differ widely and may relate to health inequalities. Previous studies have not reported on the demographic or practice features of funding distribution for these schemes.^6,10^ Given the difficulties in gaining research access to local scheme data, it was aimed to confine the study to the distribution of national schemes and to determine the extent to which DES funding was equitably distributed.

## Method

### Design & setting

A cross-sectional analysis was conducted of data from all general practices in England from 2018–2019. Descriptive data were obtained from the General and Personal Medical Services (PMS) database.^12^ Data included the demographic characteristics of patients registered at each practice such as age, sex, ethnic group, and social deprivation. Ethnic group data, derived from national census data, categorised into five broad ethnic groups,^13^ and deprivation data based on Index of Multiple Deprivation (IMD)-2015 scores, were both based on weighted mean values for the lower super output area (LSOA) of residence of all registered patients at each practice.^14^ Practice variables included the number and age distribution of registered patients, number of full-time equivalent (FTE) GPs and practice nurses, post-graduate training practice status, region, and contract type (General Medical Services [GMS], PMS, or Alternative Provider Medical Services [APMS]).

The primary outcome was to identify the determinants of DES activity in terms of practice and population characteristics. DES activity, the provision of DES, was quantified in terms of funding (£) per registered patient. DES funding was considered first as total funding and second as funding categorised according to each service provided.

### Sample

All practices were included in the initial sample (*n* = 6873). Atypical practices were excluded if they had negative funding amounts (*n* = 4) or ≤750 registered patients (*n* = 20) following a previously used method.^15^ The final sample consisted of 6849 practices.

### Statistical methods

Multiple linear regression models were constructed to determine the association between DES activity (quantified in terms of £/patient), and the practice level predictor variables. The unstandardised regression coefficient (B) was used to calculate the change in DES funding (£/patient) for a unit change in the predictor variable. Region was included in the model as a covariate; region and clinical commissioning group (CCG) were also added to a multi-level model in a sensitivity analysis to further explore the association with social deprivation. Explanatory variables were excluded if they displayed collinearity, with a variance inflation factor (VIF) >10. Following analysis of total DES activity, further models were constructed to explore the association between individual components of DES activity, social deprivation, and ethnic group, adjusted for the same covariates included in the original model. Separate regression models were constructed for each of the 10 DES components as outcome variables. All statistical analysis was undertaken using Stata software (version 14).

## Results

The mean DES funding per registered patient was £6.25 (standard deviation [SD] = £2.58; 10^th^ centile = £3.41; 90^th^ centile = £8.83). Mean DES payments were lower in the most deprived compared with least deprived LSOA quintiles at £5.96 and £6.55, respectively (*t* = –6.16, *P*<0.001).

DES activity was offered by 99.9% of practices. The numbers and proportion of practices offering each of the 10 DES components are summarised in Table 1.

The factors associated with DES funding are displayed in Table 2. The regression model accounted for 23.3% of the variation (adjusted *R* ^2^) in DES funding. The following explanatory variables were excluded on the basis of a VIF >10: LIS payments and GP contract type (GMS, PMS, or APMS). VIF values for included variables were all <5.0. Post-estimation checks, including P-P and Q-Q plots, confirmed that the analysis met the homoscedasticity and normality assumptions of linear regression.

**Table 2.** Demographic and practice determinants of DES funding (£/patient): adjusted regression model

**Predictor variable**	**DES funding model: regression coefficient, B (95%** CI)
Practice list size (per 10 000 registered patients)	0.44 (0.32 to 0.56)^a^
Single-handed practice	–0.01 (–0.26 to 0.24)
Practice staffing (number of staff per 10 000 registered patients)	
GPs	0.09 (0.07 to 0.12)^a^
Practice nurses	0.15 (0.11 to 0.19)^a^
Administrative staff	0.01 (–0.01 to 0.03)
Direct patient care staff (for example, clinical pharmacists)	0.08 (0.05 to 0.11)^a^
Age of registered patients, years	
0–4	33.2 (26.0 to 40.4)^a^
5–14	–0.49 (–4.33 to 3.36)
45–64	7.49 (5.22 to 9.75)^a^
≥65	3.30 (1.38 to 5.23)^b^
Social deprivation quintiles of registered patients	
Deprivation quintile 1 (least deprived)	ref
Deprivation quintile 5 (most deprived)	–0.35 (–0.60 to –0.10)^b^
Deprivation quintile 4	–0.09 (–0.30 to 0.13)
Deprivation quintile 3	–0.03 (–0.22 to 0.17)
Deprivation quintile 2	0.02 (–0.16 to 0.21)
Ethnic group of registered patients	
Black	–1.45 (–2.92 to 0.02)
Asian	0.65 (0.07 to 1.23)^c^
Region	
East of England	ref
London	–0.30 (–0.58 to –0.02)^c^
Midlands	0.24 (0.01 to 0.47)^c^
North East and Yorkshire	0.49 (0.24 to 0.73)^a^
North West	0.19 (–0.07 to 0.44)
South East	0.003 (–0.24 to 0.25)
South West	0.81 (0.32 to 1.10)^a^
Practice income (£/100 patients)	
Capitation payments	–0.01 (–0.02 to –0.01)^a^
QOF payments	0.15 (0.13 to 0.18)^a^
MPIG payments	0.01 (–0.14 to 0.11)

Assocations with each predictor are adjusted for the effect of all other predictors in the Table. ^a^significant, *P*<0.001. ^b^significant, *P*<0.01. ^c^significant, *P*<0.05. DES = directed enhanced services. QOF = Quality and Outcomes Framework. MPIG = minimum practice income guarantee.

DES payments to practices in the most deprived quintile were lower than those in the least deprived quintile: –£0.35 (95% CI = –£0.60 to –£0.10) per patient. Although DES payments were also lower in the third and fourth most deprived quintiles, the association was not significant. Higher DES payments were associated with higher GP staffing levels, £0.09 (95% CI = £0.07 to £0.12), nurse staffing, £0.15 (95% CI = £0.11 to £0.19), or other clinical staff, £0.08 (95% CI = £0.05 to £0.11); all values expressed per full-time equivalent, per 10 000 patients.

There were also differences in DES funding according to the age distribution of registered patients and region (Table 2). Higher DES funding was associated with a higher proportion of children aged 0–4 years. Higher DES funding was also associated with other practice funding sources with higher QOF payments but with lower capitation fees. In the sensitivity analysis, based on a more detailed multi-level analysis of the effects at region and CCG level, there were small differences in regression coefficients when compared with the original model, but no changes in the significance of predictor variables (data not shown).

Further modelling of demographic and practice determinants was conducted for individual components of DES activity. Analysis included all predictor variables contained in the primary analysis but for simplicity only the associations with social deprivation, ethnic group, and age characteristics are summarised in Table 3. In deprived communities, lower DES funding was seen for all immunisations (five categories), as well as for extended hours access. For example, DES funding for influenza and pneumococcal vaccination was lower in the most deprived compared with least deprived quintile: –£0.15 (95% CI = –£0.21 to –£0.08). Learning disability checks was the only DES activity to be positively associated with social deprivation.

**Table 3.** Demographic and practice determinants of individual components of DES funding (£/patient): adjusted regression model

**Predictor variable**	**Influenza & pneumococcal immunisation**	**Rotavirus & shingles immunisation**	**Meningitis immunisation**	**Childhood immunisations**	**Pertussis immunisation**	**Learning disabilities checks**	**Minor surgery**	**Extended hours access**	**Violent patient services**	**Out of area, in-hours urgent care**
Adj. *R* ^2^: 0.50	Adj. *R* ^2^: 0.29	Adj. *R* ^2^: 0.39	Adj. *R* ^2^: 0.19	Adj. *R* ^2^: 0.21	Adj. *R* ^2^: 0.11	Adj. *R* ^2^: 0.09	Adj. *R* ^2^: 0.08	Adj. *R* ^2^: 0.09	Adj. *R* ^2^: 0.002
Age of registered patients, years (comparator: 15–44)	Regression coefficient, B (95% CI)									
0–4	0.48(–1.29 to 2.25)	1.78^a^(1.58 to 1.98)	5.24^a^(4.95 to 5.53)	18.7^a^(16.5 to 20.9)	0.98^b^(0.89 to 1.08)	0.09(–0.98 to 1.15)	1.37(–2.95 to 5.69)	2.69(–0.32 to 5.71)	1.39(–1.82 to 4.61)	–0.01(–0.07 to 0.04)
5–14	0.77(–0.18 to 1.71)	–0.22^a^(–0.33 to –0.11)	–0.72^a^(–0.88 to –0.57)	1.65^b^(0.47 to 2.83)	–0.19^a^(0.24 to –0.14)	0.80^b^(0.23 to 1.37)	–0.004(–2.31 to 2.30)	0.46(–1.14 to 2.06)	–3.04^b^(–4.75 to –1.33)	–0.0004(–0.03 to 0.03)
45–64	0.69^c^(0.14 to 1.24)	0.04(–0.02 to 0.10)	–0.09(–0.18 to 0.01)	1.95^a^(1.26 to 2.65)	0.02(–0.11 to 0.06)	0.24(–0.09 to 0.57)	2.05^b^(0.69 to 3.40)	1.36^b^(0.42 to 2.30)	1.35^b^(0.35 to 2.35)	0.007(–0.02 to 0.01)
≥65	5.71^a^(5.24 to 6.18)	0.54^a^(0.49 to 0.60)	–0.30^a^(–0.38 to 0.22)	–0.79(–1.38 to –0.20)	–0.09(–0.11 to –0.06)	–0.37^c^(–0.65 to –0.08)	2.17^a^ (1.01 to 3.32)	–1.02^c^(–1.82 to –0.21)	–2.43^a^(–3.29 to –1.58)	–0.003(–0.02 to 0.01)
Social deprivation (most deprived compared with least deprived quintile)	–0.15^a^(–0.21 to –0.08)	–0.02^a^(–0.03 to 0.02)	–0.03^a^(–0.04 to –0.02)	–0.12^b^(–0.20 to –0.05)	–0.01^a^(–0.02 to –0.01)	0.10^a^(0.07 to 0.14)	0.11(–0.04 to 0.26)	–0.24^a^(–0.35 to –0.14)	0.03(–0.08 to 0.14)	–0.001(–0.003 to 0.001)
Black ethnic group (compared with White ethnic group)	0.08(–0.28 to 0.44)	–0.02(–0.06 to 0.02)	–0.02(–0.08 to 0.04)	–1.09^a^(–1.54 to –0.64)	–0.04^a^(0.06 to –0.01)	–0.26^c^(–0.47 to –0.04)	–0.66(–1.54 to 0.22)	0.82^b^(0.20 to 1.43)	–0.45(–1.10 to 0.21)	0.001(–0.01 to 0.01)
Asian ethnic group (compared with White ethnic group)	0.37^a^(0.23 to 0.52)	–0.01(–0.02 to 0.01)	0.01(–0.01 to 0.04)	0.08(–0.10 to 0.26)	–0.02(–0.01 to 0.02)	0.07(–0.02 to 0.15)	0.16(–0.19 to 0.51)	0.06(–0.19 to 0.30)	–0.03(–0.23 to 0.29)	0.002(–0.002 to 0.006)

The B coefficients displayed are adjusted for all predictors included in the original regression model, Table 2. For simplicity, only age, deprivation, and ethnic group predictors are shown in this Table.

Adjusted *R*2 values refer to values for the regression model for each individual directed enhanced services (DES) component. ^a^significant, *P*<0.001. ^b^significant, *P*<0.01. ^c^significant, *P*<0.05.

In practices with a higher proportion of Black patients, there was less childhood immunisation activity, although extended hours access activity was higher in these areas. In Asian communities, the only significant association was with higher levels of influenza and pneumococcal immunisation activity.

## Discussion

### Summary

National provision of additional primary care services in the form of DES accounts for just over 4% of practice funding. For a practice with 10 000 registered patients, mean DES funding amounted to £62 500 (2018–2019). DES payments were lower in practices serving deprived communities. Based on the authors' model adjusting for demographic and practice factors, a practice of 10 000 patients in the most deprived quintile could expect DES payments to be £3500 lower than a practice of 10 000 patients in the least deprived quintile. More severe levels of social deprivation are associated with higher healthcare needs.^16^

When individual components of DES were considered in the secondary analysis, provision of all five immunisation components was lower in deprived communities, as was provision of extended hours access. For example, in practices in the most deprived quintile, modelled payments for influenza and pneumococcal immunisation would be £1500 lower, for provision of extended hours access would be £2400 lower, and for provision of childhood immunisations would be £1200 lower, when compared with practices in the least deprived quintile (figures extrapolated for 10 000 registered patients). Immunisation rates are known to be lower in deprived communities^17^ and the DES income shortfall is likely to contribute to this deficit and the associated health inequalities. Extended hours access has been associated with reductions in A&E attendance, but lack of provision in deprived communities may be a factor accounting for higher A&E attendance rates in these areas.^18,19^ Not all DES displayed this pattern of funding shortfalls in deprived communities: in the above model, practices in the most deprived quintile would obtain £1000 more funding than practices in the least deprived quintile for learning disability health checks.

Findings related to ethnic group were mixed. Overall DES provision was not significantly related to Black ethnic group and, indeed, was significantly higher in practices with a larger proportion of Asian patients. Some individual components were less well provided in practices with a high proportion of Black patients: learning disability health checks and both childhood and pertussis immunisation. In a systematic review of interventions to reduce immunisation inequalities, successful interventions in deprived, multi-ethnic communities were those that increased in intensity, targeting persistent non-responders.^17^ DES underprovision, although a single intervention, may contribute to immunisation inequalities in the Black community.

Not all services followed this pattern of health inequality. Learning disability checks were more commonly provided in deprived areas; extended hours access was more common in areas with a higher proportion of Black patients; and influenza and pneumococcal immunisation was more commonly provided in areas with a higher proportion of Asian patients, which is likely to represent immunisation of a mainly older population.

DES provision was associated with specific age groups and regions. Substantially higher funding in the 0–4 and ≥65-year age range is likely to reflect immunisation provision directed to specific age cohorts. For example, rotavirus and shingles, meningitis, and childhood immunisations are all associated with higher coefficients in the 0–4-year-old age group, the likely age group for the intended DES activity. Similarly, minor surgery is associated with the highest coefficient in the ≥65-year age group, the likely main beneficiary for primary care minor surgery. Small but significant regional differences meant that the North East and South West regions had higher levels of DES activity with London relatively underprovided, even after adjustment for possible confounding. Known higher health needs in the north of England^9^ may be reflected in greater DES activity in the North East, but the North West does not benefit from significantly higher DES activity.

Primary care structures were associated with DES provision. Larger practices with higher GP, practice nurse, and other clinical staffing levels relative to the number of registered patients had higher DES activity, implying that well-staffed practices were better able to deliver DES activity. Other studies have suggested that higher practice staffing levels translate into higher achievement of quality outcomes.^20^ Higher QOF income and achievement was associated with higher DES provision, which may reflect practice attributes aligned to successful public health target achievement. Capitation payments, which are based on a modified Carr-Hill formula, are designed to be higher in areas of higher healthcare need; the negative relationship between DES and capitation payments implies that DES activity was lower where healthcare need was higher, adding to the evidence that the DES provision may contribute to health inequalities.

### Strengths and limitations

This study is the first to explore the equity of additional service provision, which, since recent reductions in the size of the QOF, now accounts for a substantial part of non-capitation fee-related practice income. Use of a national dataset has ensured the generalisability of findings. The model was only able to explain about a quarter of the variation in service provision implying that factors not contained within national datasets are contributing to variation in DES activity, although the relation between these other factors and population health needs cannot be determined. Data on ethnic group and deprivation are derived from small area level data, much in turn derived from the 2011 national census, and may not apply to individually registered patients at any given practice. Analysis of social deprivation data were conducted using quintiles of deprivation, which removed the effect of outliers, but may have resulted in underestimates of the association between DES payment and extremes of deprivation or affluence. Some of the DES funding inequalities identified may be attenuated by LIS schemes, but the authors are unable to account for this owing to lack of data on LIS funding structures. Finally, associations in cross-sectional data may imply causality but need to be repeated over time in order to strengthen causal inferences.

### Comparison with existing literature

Other published studies have noted an inverse association between overall enhanced services funding and social deprivation, consistent with the present study's findings.^7,10^ Both studies considered aggregate enhanced services, not distinguishing between DES and LIS services, nor exploring the distribution of individual components of the enhanced services. The findings of the present study provide more precision about demographic and practice organisational features associated with variation in the individual components of enhanced service activity.

Health improvement schemes may end up widening health inequalities as a result of differential uptake by different social groups or differential provision by healthcare providers,^21^ although the authors of this study did not find that the health inequality gap widened progressively over time. These consequences may be unintended but are highly predictable. Equality of funding (‘flat funding’) in the presence of unequal healthcare needs simply widens healthcare inequality.^21^ Without additional incentives, or ‘positive discrimination’, there will inevitably be a shortfall between healthcare provision and healthcare need in deprived communities.^21^

### Implications for practice

The findings have focused on the mismatch between one aspect of primary healthcare provision and societal patterns of social deprivation and ethnic group. The mismatch raises questions about the funding of primary care and health equity. Resource utilisation is suboptimal in UK primary care.^7,9^ At present, only capitation payments are weighted for estimates of population healthcare needs. A fairer resource allocation might be achieved by weighting both primary care performance payments and capitation payments according to estimates of population need.^21^ Alternatively, enhanced services themselves could be designed to provide enhanced care tailored to the needs of socially deprived^22^ or ethnically diverse communities.^3^ Indeed, recent enhanced services proposals include a DES termed ‘tackling neighbourhood inequalities’ as part of the primary care network DES for implementation in 2021.^5^ The DES proposes practical approaches to reducing health inequalities within localities, although it is likely to offer equal funding to all localities with no opportunity to fund more radical investment in localities characterised throughout by high levels of social deprivation and healthcare need.
